# AnemiaScore: An Investigator-Developed Rule-Based Clinical Decision-Support Framework for Cancer-Related Anemia—A Single-Center Retrospective Proof-of-Concept Concordance Study

**DOI:** 10.3390/diagnostics16172796

**Published:** 2026-08-31

**Authors:** Antonio Macciò, Gianuario Sanna, Manuela Neri, Paolo Albino Ferrari

**Affiliations:** 1 Department of Obstetrics and Gynecology and Gynecological Oncology, Azienda di Rilievo Nazionale ed Alta Specializzazione “G. Brotzu”, Piazza A. Ricchi 1, 09121 Cagliari, Italy; antoniopm.maccio@aob.it (A.M.); manuela.neri@aob.it (M.N.); 2 Department of Oncological Surgery, Azienda di Rilievo Nazionale ed Alta Specializzazione “G. Brotzu”, Piazza A. Ricchi 1, 09121 Cagliari, Italy; 3 Department of Thoracic Surgery, Azienda di Rilievo Nazionale ed Alta Specializzazione “G. Brotzu”, Piazza A. Ricchi 1, 09121 Cagliari, Italy

**Keywords:** cancer-related anemia, clinical decision support, iron deficiency, intravenous iron, concordance study

## Abstract

**Background/Objectives**: AnemiaScore is an investigator-developed, deterministic, rule-based clinical decision-support framework for cancer-related anemia. This initial proof-of-concept study evaluated agreement with recorded local oncologist decisions, used as a clinical reference comparator rather than an independent standard. **Methods**: This single-center retrospective study analyzed 180 directly comparable A–C cases in the primary three-category analysis. An exploratory full-cohort analysis included 188 eligible cases and descriptively incorporated Category D decisions arising either from the five-input laboratory core or from clinical factors outside that core. **Results**: In the primary cohort, overall percent agreement was 77.8% (140/180; 95% CI, 71.2–83.2%), Cohen’s κ was 0.637 (95% bootstrap CI, 0.537–0.731), and Gwet’s AC1 was 0.680 (95% bootstrap CI, 0.588–0.767). Among eight recorded clinician-override decisions, four met the laboratory-based R0 criterion, two were classified as Category D only through the external clinical-override classification, and two were not captured. The exploratory four-category agreement was 77.7% (146/188; 95% CI, 71.2–83.0%). **Conclusions**: AnemiaScore reproduced a substantial proportion of local decision patterns, but concordance does not establish therapeutic appropriateness, clinical efficacy, safety, or superiority. Prospective multicenter validation with independent review and clinical and patient-reported outcomes is required before routine use.

## 1. Introduction

Cancer-related anemia (CRA) is a prevalent complication of cancer and anticancer treatment, affecting an estimated 40–75% of patients according to tumor type, disease stage, inflammatory burden, and treatment regimen [1,2,3]. In addition to fatigue, cognitive impairment, and reduced physical function, anemia is associated with transfusion requirements, poorer tolerance of anticancer therapy, and adverse clinical outcomes [1,2].

CRA also has organizational and supportive-care implications. Heterogeneous assessment of iron deficiency may contribute to delayed correction, undertreatment, avoidable transfusion exposure, repeated outpatient evaluations, treatment delays, and variable use of supportive-care resources. Standardized interpretation of iron indices and inflammatory markers is therefore clinically relevant, particularly in settings without immediate access to dedicated hematology or supportive-care expertise.

CRA is multifactorial and may reflect absolute iron deficiency (AID) due to blood loss, malnutrition, or impaired intake; functional iron deficiency (FID) driven by hepcidin-mediated iron sequestration during systemic inflammation; cytokine-mediated suppression of erythropoiesis; myelosuppressive treatment; renal dysfunction; or bone-marrow involvement [2,4,5,6]. The assessment should integrate hemoglobin, iron indices, and inflammatory markers rather than rely on hemoglobin alone [2,4,6].

Guidelines from the European Society for Medical Oncology (ESMO) and the National Comprehensive Cancer Network (NCCN) recommend systematic evaluation of iron status and targeted correction of iron deficiency in patients with cancer. Intravenous (IV) iron is favored in several oncologic contexts, particularly when inflammation limits intestinal iron absorption or when a rapid hematologic response is clinically desirable [3,7,8,9]. Recent reviews further emphasize that FID is common in cancer and that ferritin must be interpreted in the context of inflammation and circulating iron availability [6,9].

Although IV iron is central to guideline-based management of AID and selected FID profiles, oral approaches may have a limited, context-dependent role in carefully selected patients with mild anemia, low-grade inflammation, and no clear indication for IV iron. Lactoferrin has anti-inflammatory, immunomodulatory, antioxidant, and iron-homeostatic properties and has been studied in anemic populations and in advanced cancer [10,11,12,13,14,15]. However, lactoferrin is not established as standard-of-care treatment for CRA and should not replace guideline-based iron repletion when IV iron is clearly indicated.

Clinical decision-support systems (CDSSs) may reduce unwarranted variation, clarify decision rules, and support guideline-congruent management [16,17,18]. The web-based RESPOND system previously showed that computerized guidance could support anemia management in oncology; however, contemporary transparent tools that explicitly distinguish IV iron, a context-dependent lactoferrin option, watchful waiting, and safety-sensitive clinician reassessment remain limited [18].

To formalize this clinical reasoning, we developed a mobile-compatible, deterministic, rule-based CDSS for CRA assessment and therapeutic orientation, which we named AnemiaScore. The framework has a five-input laboratory core integrating hemoglobin (Hb), ferritin, serum iron, C-reactive protein (CRP), and fibrinogen. Core outputs are IV iron, lactoferrin as a clinician-mediated option, watchful waiting, or laboratory-triggered reassessment. In the exploratory full-cohort analysis, clinical factors outside the five-input laboratory core could prompt reassessment and supersede the laboratory output; these cases were classified descriptively rather than treated as independently generated algorithmic outputs. The cited guidance and mechanistic literature concerning inflammation-driven FID, interleukin-6, hepcidin biology, oxidative stress, and multitargeted CRA management provided the biological and clinical rationale for the framework [1,2,4,10,11,12,19,20], but did not describe or validate its final operational rule hierarchy.

The present study was designed as an initial proof-of-concept retrospective concordance assessment of this investigator-developed rule set against treatment decisions recorded by oncologists in an anonymized real-world cohort. It was not designed to establish diagnostic accuracy, autonomous clinical validity, or treatment efficacy. Its purpose was to quantify how closely the explicit rule hierarchy reproduced local decision patterns and to identify the requirements for subsequent independent, prospective, outcome-based validation.

## 2. Materials and Methods

### 2.1. Study Design

This single-center retrospective agreement study used anonymized data from consecutive adult patients with histologically confirmed malignancy and documented anemia who were treated at the Department of Medical Oncology, Oncology Hospital “Armando Businco”, Azienda di Rilievo Nazionale ed Alta Specializzazione “G. Brotzu”, Cagliari, Italy, between January 2022 and December 2024. The study was conducted in accordance with the Declaration of Helsinki and applicable institutional and national regulations. Data were fully anonymized before analysis; individual consent was waived in accordance with institutional policy for retrospective analyses of anonymized routine-care data. AnemiaScore was applied only after the treating oncologists had made and recorded their clinical decisions and therefore had no influence on patient assessment, diagnostic procedures, treatment allocation, or clinical management.

The analytic dataset consisted exclusively of real-world clinical records extracted from the institutional database. We used no simulated, synthetic, AI-generated, or artificially imputed patient-level records. Only records with all AnemiaScore input variables were included; no missing values were imputed.

### 2.2. Patient Selection

Inclusion criteria were age ≥18 years; histologically confirmed solid or hematologic malignancy; Hb <12.0 g/dL for women or <13.0 g/dL for men at the index laboratory assessment; availability of Hb, ferritin, serum iron, CRP, and fibrinogen measured within a 7-day window; and a documented treatment decision by the attending oncologist within 14 days of the index assessment.

Exclusion criteria were active hemolytic anemia; red-blood-cell transfusion within 30 days before the index assessment; current treatment with an erythropoiesis-stimulating agent (ESA); missing or ambiguous documentation of the treatment decision; and refusal of treatment for any reason.

### 2.3. Data Collection and Clinical Reference Comparator

The extracted variables were age, sex, tumor group, malignancy class, disease stage, treatment regimen, treatment intent, anonymous treating-oncologist identifier, hemoglobin, ferritin, serum iron, C-reactive protein, fibrinogen, active bleeding, renal impairment, bone-marrow involvement, a retrospectively recorded fatigue score, transfusion urgency, AnemiaScore output, and the recorded oncologist decision. The clinical decision was categorized as (A) IV iron, (B) oral lactoferrin, (C) no specific treatment/watchful waiting, or (D) clinician reassessment/override, including transfusion, erythropoiesis-stimulating-agent initiation, or further diagnostic work-up.

Treatment decisions were made during routine care by six board-certified medical oncologists before AnemiaScore outputs were generated and without access to those outputs. The six oncologists whose decisions formed the reference comparator did not participate in development of the framework. Individual oncologists contributed 19–42 cases (10.6–23.3% of the primary cohort), so no single clinician accounted for one quarter of the observations. All followed the institutional anemia-management protocol, which recommends assessment of iron status before treatment. Because each case had only one original treating-oncologist decision, this retrospective dataset could not support formal inter-oncologist agreement.

The oncologist decision was treated as a clinical reference comparator rather than an infallible gold standard. Records with missing or irreducibly ambiguous documentation were excluded. When the recorded management decision was documented but its mapping to categories A–D was uncertain, two senior oncologists classified the existing decision by consensus; this procedure did not generate a new clinical decision. Prospective documentation of reviewer independence and blinding to the AnemiaScore output was unavailable, and this local categorization process was not an independent validation standard. Category D denoted a recorded reassessment/override decision, including transfusion, ESA initiation, or further diagnostic work-up.

### 2.4. Development and Definition of the AnemiaScore Rule Set

AnemiaScore is an investigator-developed, deterministic hierarchical decision framework rather than an additive or continuous numerical score. The term “AnemiaScore” is retained as the designated name of the framework and should not be interpreted as a quantitative score. It was constructed by translating ESMO 2018 and NCCN 2024 guidance and integrating mechanistic and clinical evidence on inflammation, iron metabolism, and supportive treatment of CRA into operational decision rules [1,2,3,4,7,10,11,12,19,20]. These sources supplied the biological and clinical rationale but did not describe or validate the final hierarchy. The framework was not fully specified before the study records were examined. The final rule hierarchy was developed in the same institutional environment after investigators became familiar with the records and local practice, and it was then fixed before calculating the agreement statistics. The archived patient-level AnemiaScore output field represents that final hierarchy. During revision, we corrected the human-readable specification to reproduce the archived output mapping; we did not change any patient-level output or agreement statistic. We did not formally compare multiple candidate rule sets or optimize against the observed agreement coefficients. The six oncologists whose decisions formed the comparator were not involved in framework development. Accordingly, the study combines derivation and evaluation within the same environment and is vulnerable to derivation–evaluation bias and institutional overfitting. Supplementary Table S1 distinguishes evidence-supported concepts from investigator-defined implementation choices.

Table 1 summarizes the input variables and operational thresholds. Supplementary Figure S1 and Supplementary Tables S2 and S3 provide the complete decision flowchart, rule hierarchy, overlap resolution, and handling of borderline and safety-sensitive profiles. Rules are applied sequentially. R0 has first priority. Thereafter, AID, FID, and no-algorithm-detectable-iron-deficiency pathways are applied using explicit, non-overlapping intervals; any eligible laboratory profile not captured by an A–C rule is assigned to Category D for reassessment. The inequalities displayed in the tables are controlling, and no interpolation between thresholds is performed. AID is ferritin <30 ng/mL. FID is ferritin 30–800 ng/mL, serum iron <60 µg/dL, and active inflammation defined as CRP >10 mg/L or fibrinogen >400 mg/dL. Ferritin ≥100 ng/mL does not exclude FID when low serum iron and inflammation coexist.

Severe inflammation (CRP >50 mg/L or fibrinogen >600 mg/dL) activates R0 and generates a laboratory-core Category D reassessment output without automatic IV iron. When R0 is inactive, AID generates Category A at any eligible Hb value; FID generates Category A when Hb <10 g/dL and Category B when Hb is 10 g/dL or higher but remains below the sex-specific anemia threshold and 10 < CRP ≤20 mg/L. In profiles without algorithm-detectable iron deficiency, ferritin 100 to <150 ng/mL with serum iron ≥60 µg/dL, CRP ≤10 mg/L, and fibrinogen ≤400 mg/dL generates Category B, whereas ferritin ≥150 ng/mL with the same iron and inflammatory conditions generates Category C. Any remaining eligible profile generates Category D reassessment. Clinical factors outside the five-input laboratory core could prompt clinician reassessment and supersede an A–C laboratory output. In the exploratory full-cohort analysis, such cases were classified descriptively and were not treated as independently generated outputs of the laboratory algorithm. Category B remains a non-standard, context-dependent lactoferrin option; Category D is non-therapeutic.

### 2.5. Statistical Analysis

The primary endpoint was overall percent agreement (OPA) between the AnemiaScore output and the oncologist decision. We calculated Wilson score 95% confidence intervals (CIs) for OPA. Cohen’s unweighted κ was the primary chance-corrected coefficient. Because κ can be affected by category prevalence and marginal imbalance, we added Gwet’s AC1 as a complementary sensitivity coefficient [21]. We retained an exploratory linear weighted κ only as a secondary analysis, using the explicit weight matrix [[1, 0.5, 0], [0.5, 1, 0.5], [0, 0.5, 1]] for IV iron, lactoferrin, and watchful waiting, respectively.

We did not perform a formal a priori sample-size calculation because no prior agreement estimate was available for AnemiaScore or a directly comparable CRA framework. We determined sample size based on consecutive eligible records during the study period. We used the 95% CI around the primary κ estimate to describe precision; the study was not powered for subgroup comparisons. Future studies should calculate sample size from prespecified anticipated and minimally acceptable agreement coefficients, expected marginal category frequencies, desired CI width or statistical power, number of raters, and inflation for center/clinician clustering, missing data, and separately powered clinically important subgroups [22,23]. Larger study groups will be required to validate the present findings.

We assessed the distributional shape of continuous variables using histograms, quantile–quantile plots, and Shapiro–Wilk tests. We present approximately symmetric variables as mean ± standard deviation; skewed variables as median with interquartile range (IQR). Minimum–maximum values are also reported. For each management category, we calculated PA and NA using the CLSI EP12 agreement formulas after one-versus-all recoding: PA = 2TP/(2TP + FP + FN) and NA = 2TN/(2TN + FP + FN). We reported marginal category distributions for both the framework and comparator. We obtained 95% percentile CIs for κ, AC1, weighted κ, PA, and NA from 10,000 nonparametric patient-level bootstrap resamples with a fixed random seed of 4500732. A cluster-bootstrap sensitivity analysis resampled the six treating-oncologist clusters with replacement and was interpreted cautiously because only six clusters were available. Subgroup κ estimates by tumor site, anemia severity, and iron-deficiency phenotype were exploratory and are reported with denominators and bootstrap 95% CIs; we did not perform formal between-subgroup comparisons. We also described all 188 eligible cases in a four-category matrix after individually reviewing Category D handling. This exploratory analysis distinguished laboratory-core R0 outputs from Category D assignments arising only through the external clinical-override classification. Available characteristics of all 188 eligible complete-input cases and records excluded for a missing required laboratory variable were compared descriptively to assess potential selection bias. Analyses were performed using Stata version 13.1 (StataCorp LLC, College Station, TX, USA). The analysis emphasized estimates and CIs rather than null-hypothesis testing.

## 3. Results

### 3.1. Study Population

**Figure 1:**
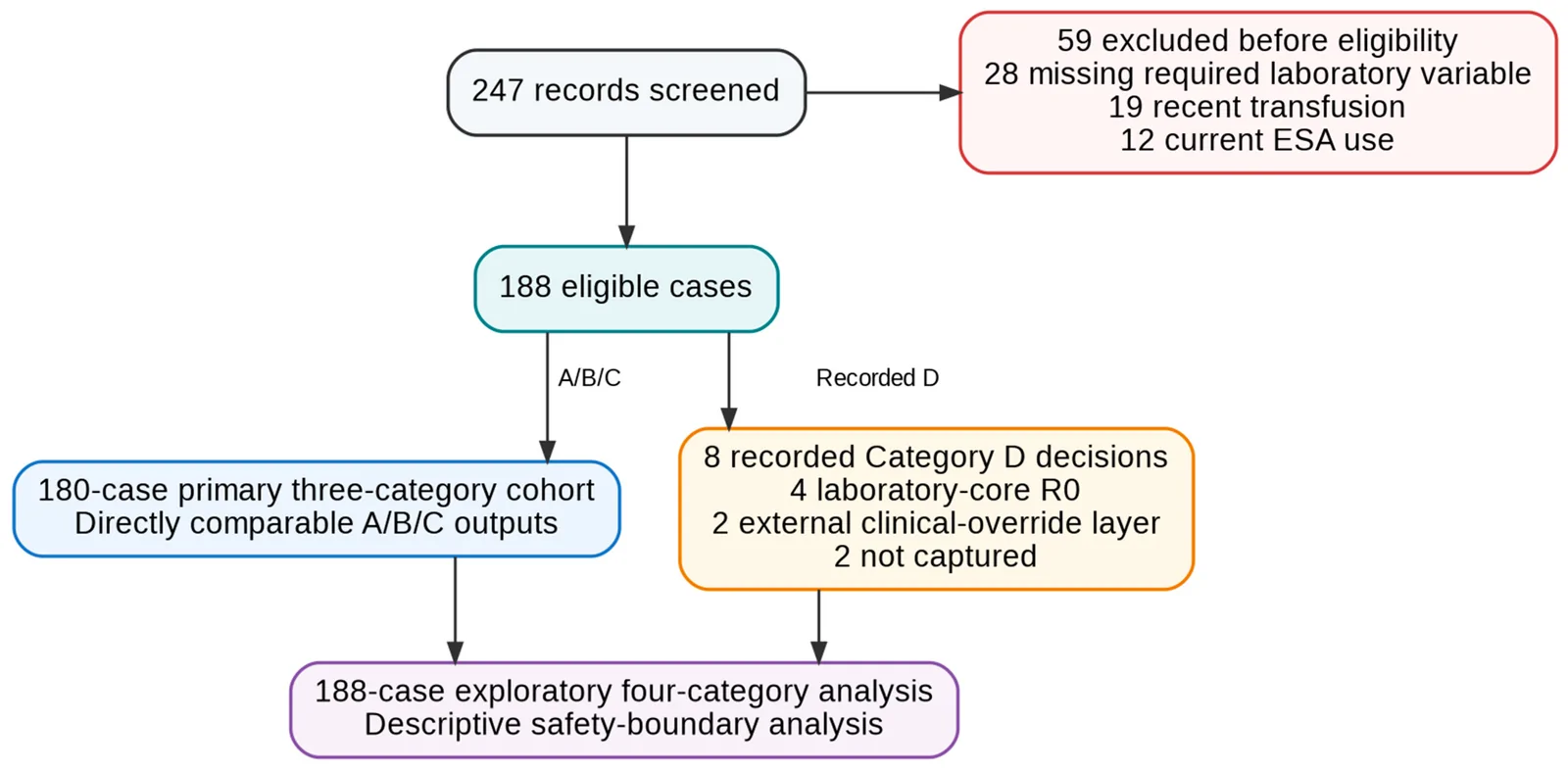
Study flow diagram. ESA, erythropoiesis-stimulating agent.

Of 247 screened records, 188 met the core clinical and laboratory eligibility criteria. Fifty-nine records were excluded before eligibility because of missing required laboratory variables (*n* = 28), recent red-blood-cell transfusion (*n* = 19), or current erythropoiesis-stimulating-agent use (*n* = 12). The directly comparable three-category primary analysis comprised 180 cases. Eight additional eligible cases had recorded clinician reassessment/override decisions and were retained for the four-category full-cohort analysis (Figure 1).

All laboratory values and clinical decision variables used in the analysis were derived from the institutional clinical database; no patient-level values were generated or simulated. Age was approximately symmetrically distributed, whereas hemoglobin, ferritin, serum iron, C-reactive protein, and fibrinogen were summarized with medians, IQRs, and ranges because several distributions were non-normal or threshold-constrained. Table 2 reports baseline characteristics.

### 3.2. Primary Agreement Analysis

The complete AnemiaScore–oncologist cross-classification is shown in Table 3. OPA was 77.8% (140/180; 95% CI, 71.2–83.2%). Cohen’s κ was 0.637 (95% bootstrap CI, 0.537–0.731), Gwet’s AC1 was 0.680 (95% bootstrap CI, 0.588–0.767), and exploratory linear weighted κ was 0.636 (95% bootstrap CI, 0.525–0.735). The framework assigned 51.1% of cases to IV iron, 23.9% to the lactoferrin option, and 25.0% to watchful waiting; the corresponding oncologist marginal proportions were 53.9%, 25.0%, and 21.1%.

Category-specific one-versus-all agreement estimates and bootstrap CIs are reported in Table 4. Agreement was highest for IV iron (PA, 85.7%; 95% CI, 80.0–90.7%; NA, 84.2%; 95% CI, 77.6–89.7%), followed by the lactoferrin option (PA, 72.7%; 95% CI, 60.9–82.6%; NA, 91.2%; 95% CI, 87.4–94.4%) and watchful waiting (PA, 65.1%; 95% CI, 51.5–75.9%; NA, 89.5%; 95% CI, 85.5–93.1%).

Discordance was multidirectional. The largest single off-diagonal flow was framework watchful waiting to oncologist IV iron (13 cases). Additional prominent flows were framework IV iron versus oncologist lactoferrin (8 cases) and framework lactoferrin versus oncologist watchful waiting (8 cases), followed by framework watchful waiting versus oncologist lactoferrin (5 cases); the remaining two directions each contained 3 cases. Lactoferrin-versus-observation disagreement remained clinically informative in low-grade inflammatory profiles, but it was not the sole source of discordance. No patient in the primary three-category cohort met the defined R0 severe-inflammation threshold; therefore, R0 performance and safety could not be evaluated in that cohort.

### 3.3. Subgroup and Sensitivity Analyses

Exploratory subgroup estimates are summarized in Table 5. By anemia severity, κ was 0.576 (95% CI, 0.433–0.703) for mild anemia (*n* = 98) and 0.430 (95% CI, 0.186–0.653) for moderate anemia (*n* = 67). All 15 severe-anemia cases were classified as IV iron by both the framework and the oncologist (OPA, 100%); κ was not estimable because neither rater used more than one category. These findings should not be interpreted as comparative evidence of subgroup performance.

By iron-deficiency phenotype, OPA was 84.7% in absolute iron deficiency (*n* = 72), 85.5% in FID (*n* = 55), and 60.4% when no algorithm-detectable iron deficiency was present (*n* = 53). In the absolute-iron-deficiency subgroup, the framework output was invariant (all category A), yielding κ = 0 despite high observed agreement; this illustrates the prevalence and marginal-distribution limitation of κ. Kappa was 0.732 (95% CI, 0.567–0.891) for FID and 0.218 (95% CI, 0.029–0.406) for no algorithm-detectable iron deficiency.

By tumor group, κ was 0.596 (95% CI, 0.419–0.755; *n* = 61) for gynecologic tumors, 0.631 (95% CI, 0.412–0.830; *n* = 40) for colorectal cancer, 0.520 (95% CI, 0.265–0.755; *n* = 32) for lung cancer, and 0.778 (95% CI, 0.601–0.926; *n* = 47) for other malignancies. These analyses were exploratory; no interaction test or claim of equivalence was retained.

Individual review of the eight Category D decisions showed that four met the five-input R0 severe-inflammation criterion. Two additional cases were assigned to Category D only after application of the external clinical-override classification for urgent management, whereas two override decisions were not captured by either the laboratory core or the applied override classification. The resulting complete four-category matrix contained 146 concordant cases among 188 (OPA, 77.7%; 95% CI, 71.2–83.0%), Cohen’s κ of 0.650 (95% bootstrap CI, 0.554–0.738), and Gwet’s AC1 of 0.716 (95% bootstrap CI, 0.638–0.793). Because two concordant D assignments depended on clinical information outside the five laboratory inputs, this full-cohort result is descriptive and must not be interpreted as an independent validation of the laboratory core. The full matrix and case classification are reported in Supplementary Tables S5 and S6. The cluster-bootstrap 95% CI for the primary κ was 0.536–0.734, and leave-one-oncologist-out estimates ranged from 0.614 to 0.677, indicating that no single clinician determined the overall result.

## 4. Discussion

This initial proof-of-concept retrospective concordance study found moderate-to-substantial agreement between the investigator-developed AnemiaScore framework and local oncologist decisions in CRA management. In the primary three-category analysis, OPA was 77.8%, Cohen’s κ was 0.637, and Gwet’s AC1 was 0.680. In the exploratory 188-case analysis, four recorded override decisions activated the laboratory-core R0 gate, two additional D assignments arose only through the external clinical-override classification, and two were not captured. The resulting four-category OPA was 77.7%, but this hybrid analysis is descriptive rather than an independent performance estimate of the five-input laboratory core. The findings do not constitute independent clinical validation.

The study must be interpreted as an agreement and feasibility evaluation, not as an efficacy trial or a validation of autonomous decision-making. The comparator was real-world oncologist practice, which may vary with symptoms, comorbidities, treatment intent, logistics, clinician experience, and institutional protocols. We did not perform an independent post hoc expert-panel review. Such a review could have provided an additional comparator, but it would not replace a prospectively specified, blinded, guideline-based adjudication process. Consequently, agreement cannot demonstrate therapeutic appropriateness, improved hemoglobin response, fewer transfusions, better quality of life, or superior safety.

Because the framework was developed and evaluated within the same institutional environment and was not fully specified before examination of the study records, the observed agreement may partly reflect derivation–evaluation bias and shared local assumptions regarding iron assessment, infusion practice, blood-product use, and supportive care. The final hierarchy was fixed before calculation of the agreement statistics and was not optimized against the resulting coefficients, but this does not remove the risk created by familiarity with the records and local practice. The six oncologists whose decisions formed the reference comparator were not involved in framework development; nevertheless, all worked within the same institutional system. External centers with different protocols, patient mixes, resource constraints, or thresholds for transfusion and IV iron may obtain different agreement estimates.

The results are broadly consistent with the limited literature on computerized support for cancer-associated anemia. RESPOND, a web-based system based on EORTC supportive-care guidance, demonstrated concurrent validity and supported guideline-congruent anemia management in patients receiving erythropoiesis-stimulating proteins [18]. The AnemiaScore framework differs by explicitly classifying IV iron, a context-dependent lactoferrin option, watchful waiting, and clinician-override safety scenarios, with investigator-defined operational rules for AID, FID, and inflammatory burden.

Agreement was highest for IV iron. This finding is relevant to the interpretation of the observed concordance because IV-iron decisions require integration of iron status, inflammatory context, anemia severity, urgency, and safety. Current guidance and clinical evidence emphasize that iron deficiency is frequent in cancer, FID is common during inflammation, and IV iron can improve hematologic outcomes in selected cancer- and chemotherapy-associated anemia settings [3,6,8,9,24,25]. The AnemiaScore framework translates part of this reasoning into traceable investigator-defined rules while preserving clinician oversight.

Discordance was multidirectional. The largest off-diagonal flow was framework watchful waiting versus oncologist IV iron (13 cases), while IV iron versus lactoferrin and lactoferrin versus watchful waiting each accounted for 8 cases. The lactoferrin–observation pattern remains clinically informative because Category B is non-standard and evidence is least directive in borderline profiles, but it should not be presented as the sole or principal source of disagreement. Lactoferrin was retained as a separate category because it was an explicitly documented local management choice; combining it with generic reassessment would conceal the direction of disagreement. Evidence directly involving patients with advanced cancer includes a randomized study of lactoferrin combined with rHuEPO-beta and related cancer-supportive-care investigations [10,11,12], whereas part of the broader evidence derives from other anemic populations and mechanistic reviews [13,14,15]. Category B is therefore exploratory and clinician-mediated and must not be presented as equivalent to guideline-supported IV iron.

No patient in the primary three-category cohort met the investigator-defined severe-inflammation threshold of CRP >50 mg/L or fibrinogen >600 mg/dL; therefore, R0 performance could not be evaluated in the primary analysis. Four patients in the complete 188-case–cohort met R0 and received a reassessment output, but this very small number cannot establish safety or validate the threshold. R0 should be understood as a precautionary reassessment gate, not as an absolute contraindication or an autonomous alternative-treatment recommendation. Patient-specific urgency and the need for transfusion, ESA therapy, or additional diagnostic work-up remain clinical decisions [2,4,5,6].

The restricted input set is pragmatic but limits etiologic discrimination. Ferritin and serum iron without transferrin saturation, transferrin or total iron-binding capacity, reticulocyte indices, vitamin B12 and folate, or formal hemolysis assessment cannot distinguish all mechanisms of CRA. In the study database, active bleeding was recorded in 7/180 cases, renal impairment in 24/180, and bone-marrow involvement in 6/180; the decision rules did not use these factors. A retrospective fatigue score was available, but it was not a prespecified validated patient-reported outcome, and formal quality-of-life data were unavailable. AnemiaScore should therefore be viewed as laboratory-pattern decision support rather than a comprehensive diagnostic pathway. Clinically relevant omitted factors should trigger reassessment or override.

The Category D analysis illustrates both the framework’s boundary and the unavoidable overlap between evaluating omitted variables and including complex override cases. The five-input R0 gate identified four of eight override decisions. Two additional cases were classified descriptively as Category D on the basis of clinical factors outside the five-input laboratory core, while two override decisions were not captured. The full four-category analysis therefore combines a laboratory-core output with clinician-derived safety information and is descriptive rather than an independent validation analysis. This distinction is necessary to present all 188 eligible cases without implying that the five laboratory inputs encode urgency, bleeding, or alternative etiologies. Prospective validation should prespecify mandatory override variables and independently adjudicate whether each reassessment flag is clinically appropriate.

Cohen’s κ can be affected by category prevalence and marginal imbalance. The revised analysis therefore reports complete marginal distributions and Gwet’s AC1 in addition to unweighted κ. The exploratory weighted κ remains secondary because IV iron, lactoferrin, and watchful waiting are clinically distinct and do not form a universally validated ordinal scale. The exact weight matrix is now reported; unweighted κ, AC1, and category-specific PA/NA remain the more directly interpretable agreement measures.

Limitations include the retrospective design; use of local oncologist decisions rather than an independent guideline-based standard; lack of full pre-specification before record examination, with consequent derivation–evaluation bias; development and evaluation within the same center with potential institutional overfitting; absence of repeated ratings needed to measure inter-oncologist agreement; lack of prospectively documented adjudicator blinding; restriction to five laboratory inputs; and absence of validated longitudinal patient-reported and clinical outcomes. The exploratory four-category analysis is additionally limited because two concordant D assignments depended on an external clinical-override classification rather than the laboratory core. Although six oncologists contributed and no clinician accounted for more than 23.3% of the primary cohort, only six clinician clusters were available. Subgroup analyses were underpowered, and κ was non-estimable or uninformative in subgroups with invariant category use. The cohort was predominantly composed of solid tumors (175/180) and chemotherapy-containing regimens (141/180), limiting inference for hematologic malignancies and other treatment contexts. Complete-input eligibility may also have introduced selection bias: compared with all 188 eligible complete-input cases, the 28 records excluded for missing laboratory data had lower hemoglobin, more moderate/severe anemia, and were less often receiving chemotherapy. Supplementary Table S7 provides detailed comparisons.

The next phase should be a preregistered, prospective multicenter study with a formally justified sample size, external investigators, an independent guideline-based adjudication panel, and repeated blinded ratings of the same cases by multiple oncologists. Inter-oncologist agreement should be measured before and after access to the framework using pairwise and multirater coefficients, category-specific agreement, and mixed-effects models accounting for clinician and center clustering. Patient-centered assessment should include baseline and longitudinal symptoms, patient preference, validated fatigue instruments such as FACIT-F or the EORTC QLQ-C30 fatigue domain, and quality of life. Clinical outcomes should include hemoglobin and iron-status response at prespecified time points, transfusion incidence and transfusion-free survival, erythropoiesis-stimulating-agent exposure, treatment delays or dose reductions, adverse events, resource use, adherence, and override frequency. Future framework versions should evaluate active bleeding, comorbidities, renal function, marrow involvement, treatment intent, and expected therapy duration as explicit inputs or mandatory reassessment triggers. Larger, separately powered tumor- and severity-specific groups are required.

## 5. Conclusions

In this initial single-center proof-of-concept assessment, AnemiaScore demonstrated moderate-to-substantial agreement with recorded local oncologist decisions in the directly comparable three-category cohort (OPA, 77.8%; Cohen’s κ, 0.637; Gwet’s AC1, 0.680). The exploratory four-category full-cohort result was numerically similar, but two concordant D assignments required an external clinical-override classification, so the estimate should be interpreted descriptively. Agreement was highest for IV iron, whereas discordance was multidirectional and included clinical factors not represented by the five-input laboratory core.

AnemiaScore should be considered an investigational, transparent, rule-based decision-support framework rather than a numerical score or stand-alone management system. Concordance with local practice does not validate the investigator-defined thresholds, establish therapeutic appropriateness, demonstrate clinical efficacy or safety, or prove superiority over standard care. Larger, prospective, multicenter validation with independent adjudication, inter-oncologist reliability, hematologic outcomes, patient-reported outcomes, and implementation endpoints is required before routine use.

## Figures and Tables

**Table 1:** AnemiaScore input variables and operational thresholds.

Variable	Unit	Clinical Reference Range	Operational interpretation in AnemiaScore
Hemoglobin (Hb)	g/dL	Women: 12–16; men: 13.5–17.5	Eligibility: Hb <12 g/dL in women or <13 g/dL in men. Explicit intervals: severe <8; moderate 8–<10; mild/borderline 10–<12; the eligible male interval 12–<13 is handled by phenotype-specific rules or default reassessment.
Ferritin	ng/mL	Women: 12–150; men: 12–300	AID: <30. FID: 30–800 when serum iron is <60 and inflammation is present. Borderline No_ID option: 100–<150 with serum iron ≥60 and low inflammation. No_ID watchful-waiting boundary: ≥150 with serum iron ≥60 and low inflammation.
Serum iron	µg/dL	Women: 60–160; men: 70–175	Low: <60; adequate: ≥60. Interpreted jointly with ferritin and inflammatory markers.
CRP	mg/L	<5	Low/no active inflammation: CRP ≤10; low-grade active inflammation: 10 < CRP ≤ 20; higher active inflammation: 20 < CRP ≤ 50; severe inflammation: CRP >50, activating R0.
Fibrinogen	mg/dL	200–400	Non-elevated: fibrinogen ≤400; moderately elevated: 400 < fibrinogen ≤ 600; severe elevation: fibrinogen >600, activating R0.

AID, absolute iron deficiency; CRP, C-reactive protein; FID, functional iron deficiency.

**Table 2:** Baseline characteristics of the primary study cohort (*N* = 180).

Characteristic	Value
Age, years, mean ± SD	62.4 ± 11.8
Age, years, median (IQR); range	62.0 (54.7–70.8); 34.1–89.2
Female sex, *n* (%)	104 (57.8)
**Malignancy class, *n* (%)**	
Solid	175 (97.2)
Hematologic	5 (2.8)
**Tumor group, *n* (%)**	
Gynecologic	61 (33.9)
Colorectal	40 (22.2)
Lung	32 (17.8)
Other	47 (26.1)
**Disease stage, *n* (%)**	
I	15 (8.3)
II	22 (12.2)
III	55 (30.6)
IV	83 (46.1)
Hematologic classification/not applicable	5 (2.8)
**Treatment regimen, *n* (%)**	
Chemotherapy alone	126 (70.0)
Immunotherapy alone	15 (8.3)
Targeted therapy alone	9 (5.0)
Chemo-immunotherapy	10 (5.6)
Chemo-targeted therapy	5 (2.8)
Supportive care/no active anticancer therapy	15 (8.3)
**Treatment intent, *n* (%)**	
Curative	81 (45.0)
Palliative	84 (46.7)
Supportive	15 (8.3)
**Anemia severity, *n* (%)**	
Mild (Hb 10–<12 g/dL)	98 (54.4)
Moderate (Hb 8–<10 g/dL)	67 (37.2)
Severe (Hb <8 g/dL)	15 (8.3)
**Iron-deficiency phenotype, *n* (%)**	
AID	72 (40.0)
FID	55 (30.6)
No algorithm-detectable iron deficiency	53 (29.4)
Hemoglobin, g/dL, median (IQR); range	10.2 (8.9–10.9); 6.3–11.9
Ferritin, ng/mL, median (IQR); range	158.2 (20.3–338.9); 6.1–649.5
Serum iron, µg/dL, median (IQR); range	40.4 (28.6–68.3); 15.3–134.0
CRP, mg/L, median (IQR); range	9.2 (4.7–18.8); 0.4–44.2
Fibrinogen, mg/dL, median (IQR); range	390 (293–485); 222–578
Active inflammation (CRP >10 mg/L), *n* (%)	89 (49.4)
Active bleeding, *n* (%)	7 (3.9)
Renal impairment, *n* (%)	24 (13.3)
Bone-marrow involvement, *n* (%)	6 (3.3)
Retrospective fatigue score, median (IQR); range	5 (4–6); 2–10
Treating oncologists, *n*	6
Cases per oncologist, range	19–42

AID, absolute iron deficiency; CRP, C-reactive protein; FID, functional iron deficiency; Hb, hemoglobin; SD, standard deviation; IQR, interquartile range. Percentages were calculated using the total primary-cohort population (*N* = 180) as the denominator and may not sum to 100% because of rounding effects.

**Table 3:** Decision matrix: AnemiaScore output versus oncologist decision (*N* = 180).

	Oncologist: IV Iron	Oncologist: Lactoferrin	Oncologist: Watchful Waiting	Total
AnemiaScore: IV iron	81	8	3	92 (51.1)
AnemiaScore: lactoferrin option	3	32	8	43 (23.9)
AnemiaScore: watchful waiting	13	5	27	45 (25.0)
Total (oncologist)	97 (53.9)	45 (25.0)	38 (21.1)	180

OPA = 77.8% (95% CI, 71.2–83.2%); Cohen’s κ = 0.637; Gwet’s AC1 = 0.680.

**Table 4:** Category-specific one-versus-all agreement and marginal distributions.

Category	AnemiaScore Output, *n* (%)	Oncologist Decision, *n* (%)	Positive Agreement, % (95% CI)	Negative Agreement, % (95% CI)
IV iron	92 (51.1)	97 (53.9)	85.7 (80.0–90.7)	84.2 (77.6–89.7)
Lactoferrin option	43 (23.9)	45 (25.0)	72.7 (60.9–82.6)	91.2 (87.4–94.4)
Watchful waiting	45 (25.0)	38 (21.1)	65.1 (51.5–75.9)	89.5 (85.5–93.1)

Positive and negative agreement were calculated separately for each category using the CLSI EP12 agreement formulas after one-versus-all recoding. Confidence intervals are percentile intervals from 10,000 patient-level bootstrap resamples.

**Table 5:** Exploratory subgroup agreement estimates.

Grouping	Subgroup	*n*	OPA, %	Cohen’s κ (95% Bootstrap CI)
Anemia severity	Mild	98	72.4	0.576 (0.433–0.703)
Anemia severity	Moderate	67	80.6	0.430 (0.186–0.653)
Anemia severity	Severe	15	100.0	Not estimable
Tumor group	Gynecologic	61	75.4	0.596 (0.419–0.755)
Tumor group	Colorectal	40	77.5	0.631 (0.412–0.830)
Tumor group	Lung	32	68.8	0.520 (0.265–0.755)
Tumor group	Other	47	87.2	0.778 (0.601–0.926)
Iron phenotype	AID	72	84.7	0.000 (0.000–0.000)
Iron phenotype	FID	55	85.5	0.732 (0.567–0.891)
Iron phenotype	No algorithm-detectable iron deficiency	53	60.4	0.218 (0.029–0.406)

OPA, overall percent agreement. Kappa was not estimable in the severe-anemia subgroup because both raters assigned all 15 cases to the same category. In the absolute-iron-deficiency subgroup, AnemiaScore assigned all cases to category A; κ = 0 therefore reflects invariant framework output rather than absence of observed agreement.

## Data Availability

The data supporting this study were derived from anonymized routine-care clinical records held in controlled institutional storage at Azienda di Rilievo Nazionale ed Alta Specializzazione “G. Brotzu”, Cagliari, Italy. The patient-level dataset is not publicly available due to privacy and institutional data governance restrictions. De-identified aggregate data are reported in the article and Supplementary Materials; additional anonymized data may be made available by the corresponding author on reasonable request, subject to institutional approval and a data-sharing agreement. The complete human-readable rule specification is provided in the Supplementary Materials. A reproducible research implementation is being developed, but an adequately documented, version-controlled, and distributable executable version is not currently available.

## Supplementary Materials

The following supporting information can be downloaded at: https://www.mdpi.com/article/10.3390/diagnostics16172796/s1, Figure S1, AnemiaScore decision flowchart; Table S1, provenance and evidentiary basis of the AnemiaScore rule components; Table S2, investigator-defined rule hierarchy and decision logic; Table S3, borderline and safety-sensitive profiles; Table S4, clinical scope and mandatory reassessment conditions; Table S5, exploratory four-category matrix in all eligible cases (*N* = 188); Table S6, patient-level review summary of the eight category D cases; Table S7, comparison of all eligible complete-input cases and records excluded for a missing required laboratory variable.

## Abbreviations

AC1, Gwet’s agreement coefficient 1; AID, absolute iron deficiency; CDSS, clinical decision-support system; CI, confidence interval; CRA, cancer-related anemia; CRP, C-reactive protein; ESA, erythropoiesis-stimulating agent; FID, functional iron deficiency; Hb, hemoglobin; IV, intravenous; NA, negative agreement; OPA, overall percent agreement; PA, positive agreement.
