# Supplementary material for: AnemiaScore: An Investigator-Developed Rule-Based Clinical Decision-Support Framework for Cancer-Related Anemia—A Single-Center Retrospective Proof-of-Concept Concordance Study
Source: Diagnostics (Basel). 2026 Aug 31;16(17):2796. doi: 10.3390/diagnostics16172796 (PMC13564862; doi:10.3390/diagnostics16172796)
Supplement: Supplementary file 1 [file diagnostics-16-02796-s001.zip › diagnostics-4500732-supplementary.pdf]

Supplementary Materials

Supplementary Figure S1. AnemiaScore Decision Flowchart.

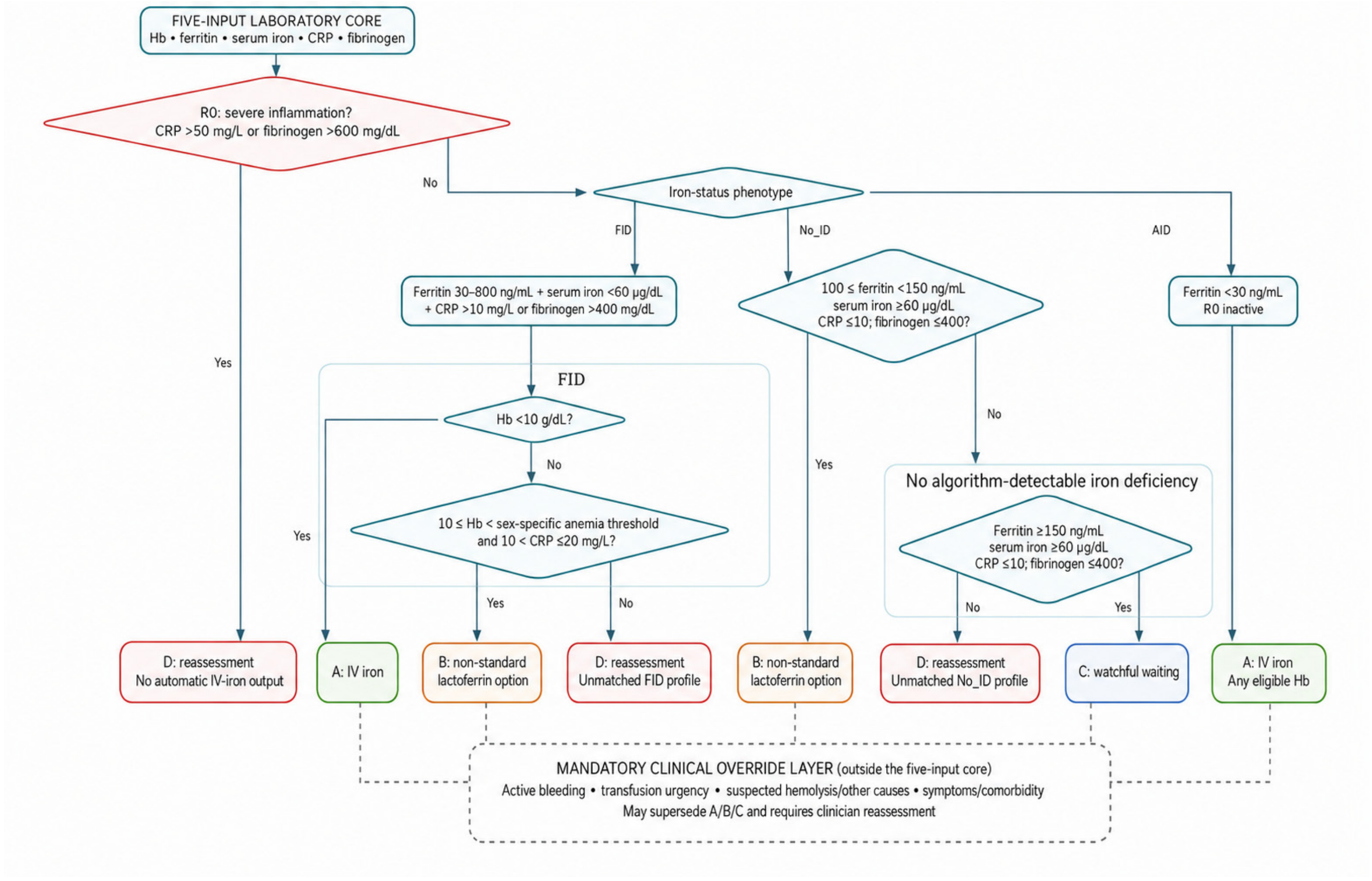

Figure S1. AnemiaScore decision flowchart. AID, absolute iron deficiency; CRP, C-reactive protein; FID, functional iron deficiency; Hb, hemoglobin; IV, intravenous; No_ID, no algorithm-detectable iron deficiency. Category B denotes a non-standard, clinician-mediated lactoferrin option. Category D may arise from the laboratory-core R0/default pathway or from a separate external clinical-override classification outside the five-input core.

Supplementary Table S1. Provenance and Evidentiary Basis of the AnemiaScore Rule Components.

AnemiaScore component	Operational implementation	Evidence-supported concept	Investigator-defined element	Supporting references
Input variables	Hb, ferritin, serum iron, CRP, and fibrinogen	Cancer-related anemia is multifactorial and should be assessed using hematologic, iron-status, and inflammatory information rather than Hb alone.	Selection of these five variables as the complete laboratory-core input set.	[1,2,4–6,20]
Absolute iron deficiency	Ferritin <30 ng/mL; Category A at any eligible Hb	Low ferritin supports depleted iron stores and absolute iron deficiency.	A single AID pathway across the eligible Hb range, its priority, and its	[3,6,7,9]

AnemiaScore component	Operational implementation	Evidence-supported concept	Investigator-defined element	Supporting references
	unless R0 or the external override layer applies		interaction with reassessment pathways.	
Functional iron deficiency	Ferritin 30–800 ng/mL, serum iron <60 µg/dL, and CRP >10 mg/L or fibrinogen >400 mg/dL	Restricted circulating iron may coexist with normal or elevated ferritin during inflammation.	The combined definition and its division into Hb <10 (A), 10 ≤ Hb below the sex-specific anemia threshold with 10 < CRP ≤20 (B), and unmatched-profile reassessment.	[2–7,9,20]
R0 severe-inflammation gate	CRP >50 mg/L or fibrinogen >600 mg/dL generates Category D reassessment	High inflammatory burden is associated with hepcidin activation, iron sequestration, and impaired iron availability.	The exact thresholds, precedence, and automatic reassessment output.	[2,4,5,20]
IV-iron pathways	AID at any eligible Hb; FID with Hb <10 g/dL, provided R0 is inactive	Guidelines and clinical evidence support intravenous iron for AID and selected FID profiles in cancer.	Exact phenotype/Hb mapping, priority, and Category A assignment.	[3,7,24,25]
Lactoferrin pathways	FID with 10 ≤ Hb below the sex-specific anemia threshold and 10 < CRP ≤20; or No_ID with ferritin 100–<150, iron ≥60, and low inflammation	Lactoferrin has iron-binding and immunomodulatory properties and has been investigated in cancer-related and other anemic populations; it is not established standard CRA treatment.	The non-overlapping Hb, ferritin, iron, and inflammatory windows and mapping to Category B.	[10,12–15]
Watchful waiting	No_ID with ferritin ≥150, serum iron ≥60, CRP ≤10, and fibrinogen ≤400	In the absence of demonstrable correctable iron deficiency, anemia may be inflammatory, disease-related, treatment-related, or multifactorial.	The exact low-inflammatory boundary and mapping to Category C.	[2–7,9]
Laboratory-core reassessment	R0 or any eligible laboratory profile not captured by A–C rules	Uncertain or safety-sensitive profiles require contextual interpretation rather than automatic treatment.	Creation of Category D as the core safety/default pathway.	[2–7,9]
External clinical-override classification	Active bleeding, transfusion urgency, alternative etiologies, symptoms, or	Urgent and alternative management depends on severity, symptoms,	A safety layer outside the five-input core; full-cohort analyses including it are descriptive.	[3,4,7–9]

AnemiaScore component	Operational implementation	Evidence-supported concept	Investigator-defined element	Supporting references
	comorbidity may supersede A–C	treatment context, and individualized risk.		
Hierarchy and output architecture	Sequential application of R0, phenotype-specific rules, default D, and a separate external override layer	Transparent rule-based CDSSs can make reasoning explicit and auditable while preserving clinician oversight.	The priority order, category structure, coded outputs, and AnemiaScore designation.	[16–18]

AID, absolute iron deficiency; CDSS, clinical decision-support system; CRA, cancer-related anemia; CRP, C-reactive protein; ESA, erythropoiesis-stimulating agent; FID, functional iron deficiency; Hb, hemoglobin; IV, intravenous. References support the underlying concepts but do not establish the exact AnemiaScore thresholds, priority order, or category mapping.

Supplementary Table S2. Investigator-Defined Rule Hierarchy and Decision Logic.

Rule	Priority	Iron-status/trigger	Hb/inflammation condition	Coded output	Biological/clinical rationale
R0	Safety—first	Any iron-status category	Any eligible Hb; CRP >50 mg/L or fibrinogen >600 mg/dL	Category D: reassessment; no automatic IV-iron output	High inflammatory burden is expected to increase hepcidin-mediated sequestration and reduce iron utilization [2,4,5,20].
R1a	High	AID: ferritin <30 ng/mL; R0 inactive	Hb <11 g/dL	Category A: IV iron	True iron depletion in CRA; IV iron is supported by guideline-based management [3,7,24,25].
R1b	High	AID: ferritin <30 ng/mL; R0 inactive	Hb ≥11 g/dL and below the sex-specific anemia threshold (<12 women; <13 men)	Category A: IV iron	AID remains the determining phenotype across the eligible Hb range [3,7,24,25].
R2	High	FID: ferritin 30–800 ng/mL + serum iron <60 µg/dL + CRP >10 mg/L or fibrinogen >400 mg/dL; R0 inactive	Hb <10 g/dL	Category A: IV iron	FID with moderate-to-severe anemia; IV iron may be considered when the R0 threshold is not met [3,7,24,25].
R4	Moderate	FID as defined above; R0 inactive	10 ≤ Hb below the sex-specific anemia threshold; 10 < CRP ≤20 mg/L	Category B: non-standard clinician-mediated lactoferrin option; observation acceptable	A biologically plausible but non-standard option in a low-grade inflammatory profile [4,10,12,13].

Rule	Priority	Iron-status/trigger	Hb/inflammation condition	Coded output	Biological/clinical rationale
R5	Moderate	No_ID: ferritin 100–<150 ng/mL + serum iron ≥60 µg/dL	Hb below the sex-specific anemia threshold; CRP ≤10 mg/L and fibrinogen ≤400 mg/dL	Category B: non-standard exploratory lactoferrin option; no mandatory treatment	Exploratory option in a low-inflammatory profile with borderline stores; observation remains appropriate [12,13].
R6	Low	No_ID: ferritin ≥150 ng/mL + serum iron ≥60 µg/dL	Hb below the sex-specific anemia threshold; CRP ≤10 mg/L and fibrinogen ≤400 mg/dL	Category C: watchful waiting	No algorithm-detectable correctable iron deficiency is identified [2–5,7].
R7	Default safety	Any eligible laboratory profile not captured by R0 or R1–R6	Any	Category D: reassessment	Unmatched combinations should not be forced into a therapeutic category; clinical reassessment is required.
External override	Outside core	Active bleeding, transfusion urgency, suspected alternative cause, symptoms, or comorbidity	Independent of laboratory interval	Category D may supersede A–C	This external clinical-override classification uses clinical information not represented by the five-input core and must be analyzed separately.

AID, absolute iron deficiency; CRA, cancer-related anemia; CRP, C-reactive protein; ESA, erythropoiesis-stimulating agent; FID, functional iron deficiency; Hb, hemoglobin; No ID, no algorithm-detectable iron deficiency. Reference numbers correspond to the main manuscript.

Supplementary Table S3. Borderline and Safety-Sensitive Profiles.

Profile	Key variables	Rule	Output/handling	Clinical interpretation
AID with non-severe inflammation	Ferritin <30 ng/mL; CRP ≤50 mg/L; fibrinogen ≤600 mg/dL; Hb below the sex-specific anemia threshold	R1a/R1b	Category A: IV iron	AID determines the core output across the eligible Hb range when R0 is inactive.
FID with Hb below 10 g/dL	Ferritin 30–800 ng/mL; serum iron <60 µg/dL; inflammation present; Hb <10 g/dL; R0 inactive	R2	Category A: IV iron	The FID pathway maps moderate-to-severe anemia to intravenous iron in the laboratory core.
FID with low-grade inflammation and higher Hb	FID; 10 ≤ Hb below the sex-specific anemia threshold; 10 < CRP ≤20 mg/L; R0 inactive	R4	Category B: non-standard clinician-mediated lactoferrin option; observation acceptable	A locally variable zone in which Category B remains exploratory and clinician-mediated.

Profile	Key variables	Rule	Output/handling	Clinical interpretation
AID or FID with severe inflammation	CRP >50 mg/L or fibrinogen >600 mg/dL; any eligible Hb	R0	Category D: reassessment; no automatic IV iron	R0 has precedence over the phenotype-specific therapeutic pathways.
No_ID with borderline ferritin	Ferritin 100–<150 ng/mL; serum iron ≥60 μg/dL; CRP ≤10 mg/L; fibrinogen ≤400 mg/dL	R5	Category B: non-standard exploratory lactoferrin option or observation	Final management is clinician-mediated; observation remains acceptable.
No algorithm-detectable iron deficiency	Ferritin ≥150 ng/mL; serum iron ≥60 μg/dL; CRP ≤10 mg/L; fibrinogen ≤400 mg/dL	R6	Category C: watchful waiting	Monitor and evaluate non-iron causes when clinically appropriate.
Unmatched laboratory profile	Any eligible combination not satisfying R0 or R1–R6	R7	Category D: reassessment	The default pathway prevents forced therapeutic classification of unrepresented or internally discordant combinations.
External clinical-override classification	Active bleeding, transfusion urgency, suspected alternative cause, symptoms, or comorbidity	External layer	Supersede A–C with Category D/clinician reassessment	This layer is outside the five-input core and must not be interpreted as a laboratory-algorithm output.

AID, absolute iron deficiency; CRP, C-reactive protein; ESA, erythropoiesis-stimulating agent; FID, functional iron deficiency; Hb, hemoglobin; No_ID, no algorithm-detectable iron deficiency.

Supplementary Table S4. Clinical Scope and Mandatory Reassessment Conditions.

Clinical condition or missing domain	Why the five-input framework is insufficient	Required handling	Future development priority
Active bleeding, hemodynamic instability, or urgent symptomatic anemia	Laboratory pattern classification cannot determine immediate transfusion or source-control needs.	External clinical-override classification: suspend A–C and require urgent clinician assessment/Category D.	Add structured bleeding and transfusion-urgency variables.
Suspected hemolysis, vitamin B12/folate deficiency, renal anemia, or marrow involvement	These etiologies are not represented by Hb, ferritin, serum iron, CRP, and fibrinogen alone.	External clinical-override classification: diagnostic work-up before disease-specific treatment.	Test reticulocyte indices, renal function, vitamin levels, hemolysis markers, and marrow-status variables.
Symptoms, performance status, patient preference, and comorbidity	These factors may alter the risk–benefit balance despite identical laboratory values.	Clinician-mediated decision; the laboratory output may be superseded.	Integrate structured symptoms, comorbidity, performance status, and preference measures.
Treatment intent, expected therapy duration, and anticancer regimen	Supportive-care goals differ across curative, palliative, cytotoxic, targeted, and immune-based treatment contexts.	Interpret only within the treatment plan; do not use as a standalone directive.	Include treatment intent, regimen, and anticipated duration in prospective models.
Hematologic malignancy or atypical anemia mechanism	Mechanisms and treatment pathways may differ substantially	Use remains exploratory pending mechanism-specific validation.	Plan separately powered disease-specific validation strata.

Clinical condition or missing domain	Why the five-input framework is insufficient	Required handling	Future development priority
	from common solid-tumor profiles.		

ESA, erythropoiesis-stimulating agent. Category D is non-therapeutic. It can be generated by the laboratory-core R0/default pathway or imposed by the external clinical-override classification.

Supplementary Table S5. Exploratory Four-Category Matrix in All Eligible Cases (N = 188).

	Oncologist A: IV iron	Oncologist B: lactoferrin	Oncologist C: watchful waiting	Oncologist D: reassessment/override	Total
AnemiaScore A: IV iron	81	8	3	1	93
AnemiaScore B: lactoferrin option	3	32	8	0	43
AnemiaScore C: watchful waiting	13	5	27	1	46
Category D: R0 or external override layer	0	0	0	6	6
Total	97	45	38	8	188

Concordant cases: 146/188. OPA, 77.7% (95% Wilson CI, 71.2–83.0%); Cohen's κ, 0.650 (95% bootstrap CI, 0.554–0.738); Gwet's AC1, 0.716 (95% bootstrap CI, 0.638–0.793). Four D assignments were generated by the five-input R0 gate and two only by the external clinical-override classification. The matrix is therefore descriptive and is not an independent validation estimate for the laboratory core.

Supplementary Table S6. Patient-Level Review Summary of the Eight Category D Cases.

Classification result	n	Clinical decision context	Source of D assignment or core output	Interpretation
Laboratory-core R0 correctly identified reassessment	4	Three urgent transfusion decisions and one further diagnostic work-up	Five-input R0: CRP >50 mg/L or fibrinogen >600 mg/dL	These are genuine laboratory-core D outputs.
External clinical-override classification D	2	Two urgent transfusion decisions	Clinical information outside the five-input core	These concordant D assignments are descriptive and cannot be credited to the laboratory core.
Override not captured	1	Urgent transfusion with active bleeding	Core output A	The five-input framework did not represent bleeding or transfusion urgency.
Override not captured	1	Further diagnostic work-up with renal impairment and marrow involvement	Core output C	The five-input framework did not represent the alternative clinical cause prompting work-up.

Each of the eight Category D cases was reviewed individually. To avoid reporting potentially identifying combinations in this small subset, the results are presented below in grouped form. Of the eight recorded Category D decisions, four met the five-input R0 criterion, two additional cases were classified as D only through the external clinical-override layer, and two were not captured. The hybrid full-cohort analysis is reported to address all eligible cases, but it must not be interpreted as performance of the five-input core alone.

Supplementary Table S7. Comparison of All Eligible Complete-Input Cases and Records Excluded for a Missing Required Laboratory Variable.

Characteristic	Eligible complete-input cohort (n = 188)	Missing-laboratory group (n = 28)	Descriptive interpretation
Age, years, mean ± SD	62.6 ± 11.8	59.1 ± 14.1	Broadly similar
Female sex, n (%)	108 (57.4)	19 (67.9)	No major descriptive imbalance
Hemoglobin, g/dL, median (IQR)	10.2 (8.7–10.9)	8.9 (8.2–10.4)	Lower hemoglobin among excluded records
Mild anemia, n (%)	100 (53.2)	8 (28.6)	Less frequent among excluded records
Moderate anemia, n (%)	67 (35.6)	15 (53.6)	More frequent among excluded records
Severe anemia, n (%)	21 (11.2)	5 (17.9)	More frequent among excluded records
Tumor group: gynecologic/colorectal/lung/other, n	63/42/34/49	9/7/7/5	Broadly similar distribution
Current chemotherapy, n (%)	149 (79.3)	10 (35.7)	Substantially less frequent among excluded records
Treatment regimen, n	Chemo alone 134; immunotherapy 15; targeted 9; combinations 15; supportive 15	Chemo alone 10; immunotherapy 9; targeted 9; combinations 0; supportive 0	Missingness differed by treatment context
Treatment intent: curative/palliative/supportive, n	81/92/15	14/14/0	Broadly similar curative/palliative balance
Missing variable pattern, n	Not applicable	Ferritin 10; serum iron 7; CRP 6; fibrinogen 5	Complete-input eligibility may introduce bias

The comparison is descriptive and is intended to characterize potential selection bias related to complete-input eligibility; it does not establish that data were missing at random.
